# CoopTFD: a repository for predicted yeast cooperative transcription factor pairs

**DOI:** 10.1093/database/baw092

**Published:** 2016-05-30

**Authors:** Wei-Sheng Wu, Fu-Jou Lai, Bor-Wen Tu, Darby Tien-Hao Chang

**Affiliations:** Department of Electrical Engineering, National Cheng Kung University, Tainan 70101, Taiwan

## Abstract

In eukaryotic cells, transcriptional regulation of gene expression is usually accomplished by cooperative Transcription Factors (TFs). Therefore, knowing cooperative TFs is helpful for uncovering the mechanisms of transcriptional regulation. In yeast, many cooperative TF pairs have been predicted by various algorithms in the literature. However, until now, there is still no database which collects the predicted yeast cooperative TFs from existing algorithms. This prompts us to construct Cooperative Transcription Factors Database (CoopTFD), which has a comprehensive collection of 2622 predicted cooperative TF pairs (PCTFPs) in yeast from 17 existing algorithms. For each PCTFP, our database also provides five types of validation information: (i) the algorithms which predict this PCTFP, (ii) the publications which experimentally show that this PCTFP has physical or genetic interactions, (iii) the publications which experimentally study the biological roles of both TFs of this PCTFP, (iv) the common Gene Ontology (GO) terms of this PCTFP and (v) the common target genes of this PCTFP. Based on the provided validation information, users can judge the biological plausibility of a PCTFP of interest. We believe that CoopTFD will be a valuable resource for yeast biologists to study the combinatorial regulation of gene expression controlled by cooperative TFs.

**Database URL:** http://cosbi.ee.ncku.edu.tw/CoopTFD/ or http://cosbi2.ee.ncku.edu.tw/CoopTFD/

## Introduction

Transcriptional regulation of gene expression is one of the major mechanisms for cells to respond to environmental and physiological changes (1, 2). This kind of regulation is usually accomplished by cooperative transcription factors (3–5). For example, the expression of NeuroD1, an essential pancreatic islet gene, is known to be regulated by two cooperative transcription factors Nkx2.2 and Ngn3 (3). Two transcription factors YY1 and E2F1 are known to cooperatively regulate the expression of p73, a protein which plays an important role in tumorigenesis (4). The cooperativity among transcription factors (TFs) enables cells to use a relatively small number of TFs in establishing the complex spatial and temporal patterns of gene expression. Therefore, identifying cooperative TFs is helpful for uncovering the mechanisms of transcriptional regulation.

With the advent of many high-throughput experimental technologies (e.g. DNA sequencing, microarrays, ChIP-chips, TF knockout experiments and protein arrays), important information of a cell can be obtained. For example, DNA sequencing can provide the DNA sequences of gene promoters. Microarrays can provide gene expression levels. ChIP-chips can provide the binding targets of a specific TF. TF knockout experiments can provide the genes affected by the knockout of a specific TF. Protein arrays can provide protein pairs which have physical interactions. The measurements from different high-throughput experimental technologies are valuable data which can be utilized to computationally identify cooperative TF pairs. Therefore, many computational algorithms have been developed to predict cooperative TF pairs by using one data source or integrating multiple data sources generated by high-throughput experimental technologies. Some algorithms used only gene expression data (6) or ChIP-chip data (7, 8). Several other algorithms integrated ChIP-chip data with gene expression data (9–13), promoter sequence data (14–16), protein–protein interaction data (17) or TF knockout data (18). Another several algorithms integrated more than two high-throughput data sources (19–23). Previous studies (24, 25) have shown that the performance of an algorithm is varied under different evaluation criteria such as the existence of physical/genetic interaction and the overlap with the benchmark set of known cooperative TF pairs.

Most existing cooperative TFs identification algorithms were applied to the model organism *Saccharomyces cerevisiae*. Different algorithms predicted different number of cooperative TF pairs ranging from a dozen to more than three thousands. These predicted cooperative TF pairs (PCTFPs) are valuable resources and provide testable hypotheses for future experimental investigation. Unfortunately, these PCTFPs were scattered in different papers and there is still no database that collects these PCTFPs from existing algorithms. This prompted us to construct Cooperative Transcription Factors Database (CoopTFD), which has a comprehensive collection of 2622 PCTFPs in yeast from 17 existing algorithms.

To help users judge the biological plausibility of a specific PCTFP of interest, CoopTFD provides five types of validation information: (i) the algorithms which predict this PCTFP, (ii) the publications which experimentally show that this PCTFP has physical or genetic interactions, (iii) the publications which experimentally study the biological roles of both TFs of this PCTFP, (iv) the common Gene Ontology (GO) terms of this PCTFP and (v) the common target genes of this PCTFP. Having these five types of validation information could help biologists pick up biologically plausible PCTFPs for further experimental investigation.

Well-established databases such as SGD (26), BioGRID (27) and IntAct (28) can provide protein pairs with physical/genetic interactions, suggesting plausible cooperative TF pairs. In contrast, CoopTFD can provide computationally predicted cooperative TF pairs which may or may not have physical/genetic interactions. Therefore, CoopTFD can generate alternative working hypotheses of the cooperative transcriptional regulation. We believe that CoopTFD will be a valuable resource for yeast biologists to study the combinatorial regulation of gene expression controlled by cooperative TFs.

## Construction and contents

### Collection of predicted cooperative TF pairs from 17 existing algorithms in the literature

In yeast, many cooperative TF pairs have been predicted by various algorithms in the literature. We collected 3755 non-redundant PCTFPs from 17 existing algorithms (see Table 1 for details). However, we found that many collected PCTFPs are not really TF pairs. Therefore, we removed the PCTFPs whose proteins are not TFs. In CoopTFD, a protein is regarded as a TF if it is annotated as a TF (activator/repressor) or a transcription co-factor in the regulation page of SGD (26). After the data processing, we obtained 2622 PCTFPs among 143 TFs (see Supplementary material, Figure S1 for a distribution of numbers of PCTFPs against number of algorithms which predict a PCTFP of interest).

**Table 1.** The list of 17 computational studies, which developed distinct algorithms to predict cooperative TF pairs by integrating multiple data sources

Authors of the algorithms	Published year	Data sources integrated	The number of identified predicted cooperative TF pairs (PCTFPs)
Banerjee and Zhang [9]	2003	ChIP-chip data and gene expression data	31
Harbison et al. [14]	2004	ChIP-chip data and promoter sequence data	94
Nagamine et al. [17]	2005	ChIP-chip data and PPI data	24
Tsai et al. [10]	2005	ChIP-chip data and gene expression data	18
Balaji et al. [7]	2006	ChIP-chip data	3459
Chang et al. [11]	2006	ChIP-chip data and gene expression data	55
He et al. [12]	2006	ChIP-chip data and gene expression data	30
Wang [19]	2006	ChIP-chip data, gene expression data and promoter sequence data	14
Yu et al. [15]	2006	ChIP-chip data and promoter sequence data	300
Elati et al. [6]	2007	Gene expression data	20
Datta and Zhao [8]	2008	ChIP-chip data	25
Chuang et al. [20]	2009	ChIP-chip data, gene expression data and promoter sequence data	13
Wang et al. [21]	2009	ChIP-chip data, gene expression data, promoter sequence data, PPI data, TF-gene documented regulation data and comparative genomic data	159
Yang et al. [18]	2010	ChIP-chip data and TF knockout data	186
Chen et al. [16]	2012	ChIP-chip data and promoter sequence data	221
Lai et al. [22]	2014	TF-gene documented regulation data, TFBS data and nucleosome occupancy data	27
Wu and Lai [23]	2015	TF-gene binding data and TF-gene regulation data	50

### Construction of five types of validation information for each PCTFP

To help users judge the biological plausibility of a PCTFP, we provide five types of validation information using various data sources, all of which were downloaded in February 2016. First, the number of algorithms which predicted this PCTFP is given. A PCTFP predicted by many algorithms has a low chance to be predicted by random. Therefore, the higher the number is, the higher the statistical confidence of this PCTFP is. Second, the number of publications which experimentally show that this PCTFP has physical or genetic interactions is given. The publications were retrieved from BioGRID database (27). Having physical or genetic interactions strengthens the confidence of the biological plausibility of this PCTFP. Third, the number of publications which experimentally study the biological roles of both TFs of this PCTFP is given. The publications were retrieved from SGD database (26). If a PCTFP is of biological significance, both TFs may well be studied in the same publication. Therefore, the higher the number is the more biological plausibility of this PCTFP is. Fourth, the common Gene Ontology (GO) terms of this PCTFP are given. The GO terms of a TF were retrieved from SGD database (26). Level of the GO term was calculated from the GO SQL file (29). If this calculation results in multiple levels, the level closest to the root is chosen. Having common GO terms provides users with strengthened evidence of the biological plausibility of this PCTFP. Finally, the common target genes of this PCTFP are provided. The target genes of a TF were retrieved from the YEASTRACT database (30). The regulatory associations between a TF and its target genes are validated by TF binding evidence, which means the experimental evidence (from band-shift, foot-printing or ChIP assay) showing that the TF binds to the promoters of its target genes. Since the biological role of a cooperative TF pair is to co-regulate the expression of a set of genes, knowing the common target genes of the two TFs of a PCTFP helps users evaluate the biological plausibility of a PCTFP.

In summary, CoopTFD provides five types of evidence (Algorithm Evidence, Physical/Genetic Interaction Evidence, Co-citation Evidence, Common GO Terms Evidence and Common Target Genes Evidence) to help users judge the biological plausibility of a PCTFP. Among them, Physical/Genetic Interaction Evidence and Common Target Genes Evidence are more informative than the other three types of evidence.

### Implementation of CoopTFD website

CoopTFD was built using a scripting language PHP and CodeIgniter framework. Python was used to do raw data processing. The processed data was stored in MySQL. The graphics of cooperative TF networks are generated using Cytoscape Web (31).

## Utility and discussion

### Database interface

**Figure 1.**
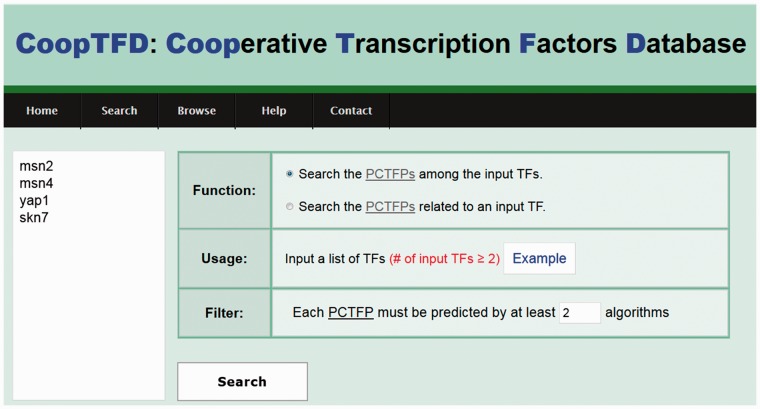
The first search mode. Users can input a list of TFs of interest and specify the lowest number of algorithms that should predict a PCTFP.

**Figure 2.**
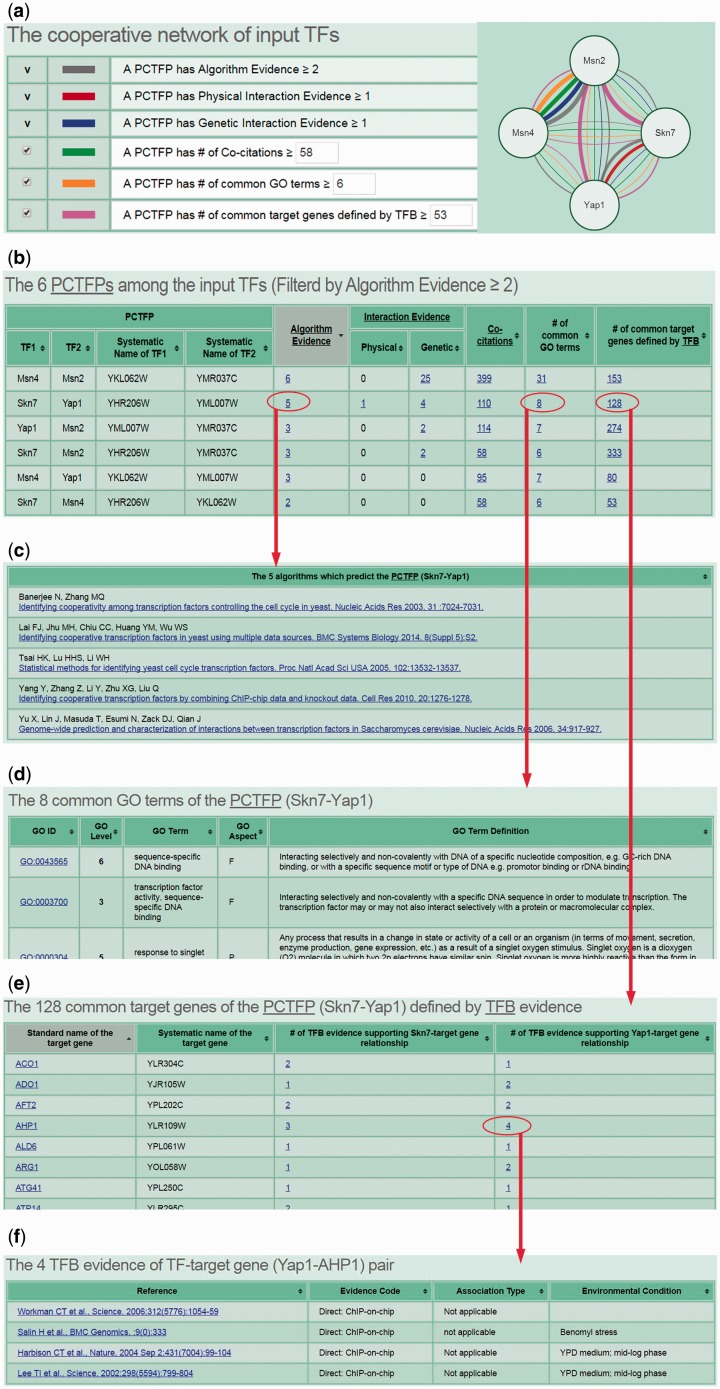
The results of the first search mode. (a) After submission, CoopTFD returns a figure showing a cooperative TF network containing all PCTFPs among the input TFs. (b) A table is given listing five types of validation information of each PCTFP in the cooperative TF network. (c) When clicking on the number in the column of ‘Algorithm Evidence’, it opens a webpage showing the details of the algorithms. (d) When clicking on the number in the column of ‘# of common GO terms’, it opens a webpage showing the names of the common GO terms. (e) When clicking on the number in the column of ‘# of common target genes defined by TFB’, it opens a webpage showing the names of the common target genes and the numbers of the TF binding (TFB) evidence that experimentally validate the TF-target gene relationship. (f) When clicking on the number in the column of ‘# of TFB evidence’, it opens a webpage showing the publications which provide the TFB evidence.

**Figure 3.**
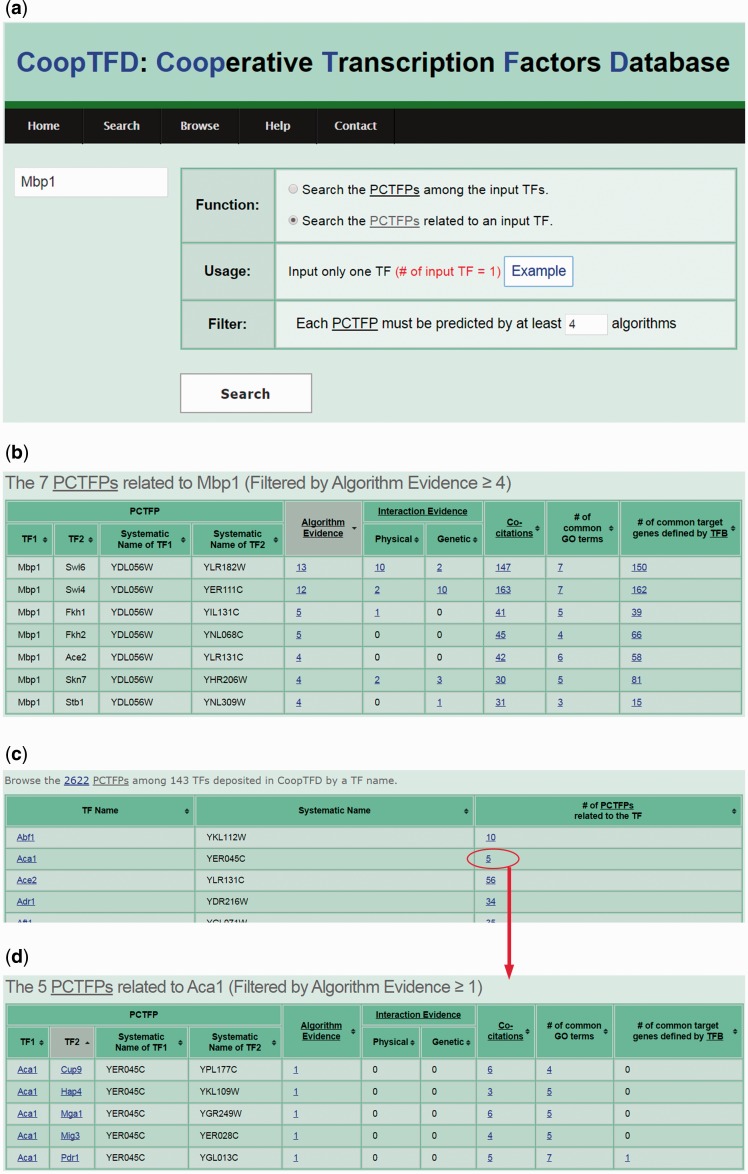
The second search mode and the browse mode. (a) In the second search mode, users can input a TF of interest and specify the lowest number of algorithms that should predict a PCTFP. (b) After submission, CoopTFD returns a table listing all PCTFPs that are related to the input TF and satisfied the specification. (c) In the browse mode, users can browse CoopTFD by a TF name. (d) When clicking on the number in the column of ‘# of PCTFPs related to the TF’, CoopTFD returns a table listing five types of validation information of each PCTFP that is related to the TF.

CoopTFD provides two search modes and a browse mode. In the first search mode, users can input a list of TFs of interest and specify the lowest number of algorithms that should predict a PCTFP (Figure 1). Then CoopTFD returns a figure showing a cooperative TF network containing all PCTFPs among the input TFs (Figure 2a). Moreover, a table is given listing five types of validation information of each PCTFP in the cooperative TF network (Figure 2b). The first three types are the number of algorithms which predict this PCTFP, the number of publications which experimentally show that this PCTFP has physical or genetic interactions, and the number of publications which experimentally study the biological roles of both TFs of this PCTFP. When clicking on the number, it opens a webpage showing the details (e.g. the authors, titles, journals and dates) of the publications (Figure 2c). The abstract of each publication in Pubmed can also be seen by clicking on the title of the publication. The fourth type of validation information is the number of common GO terms of this PCTFP. When clicking on the number, it opens a webpage showing the names of the common GO terms (Figure 2d). By clicking on the names, users will be redirected to SGD database (26) to see the details of these GO terms. The last type is the number of common target genes of this PCTFP. When clicking on the number, it opens a webpage showing the names of the common target genes and the numbers of the TF binding evidence that validate the TF-target gene relationship (Figure 2e). The publications which provide the TF binding evidence can also be shown by clicking on the number (Figure 2f). In the second search mode, users can input a TF of interest and specify the lowest number of algorithms that should predict a PCTFP (Figure 3a). Then CoopTFD returns a table listing all possible PCTFPs that are related to the input TF and satisfied the specification (Figure 3b).

In the browse mode, users can browse CoopTFD by a TF name. In total, 2622 PCTFPs among 143 TFs are deposited in CoopTFD (Figure 3c). When users click the number in the column of ‘# of PCTFPs related to the TF of interest’, our database returns a table listing five types of validation information of each PCTFP that is related to the TF of interest (Figure 3d). This is actually the same result when users select the second search mode and specify one as the lowest number of algorithms that should predict a PCTFP.

### Three scenarios of using CoopTFD

Here, we introduce three scenarios of using CoopTFD. The first scenario is as follows. If researchers have a TF of interest (e.g. Mbp1) and want to know which TFs may have cooperativity with Mbp1, they can (i) select the second search function, (ii) input Mbp1 and (iii) require that each PCTFP must be predicted by at least four algorithms (Figure 3a). After submission, CoopTFD returns seven PCTFPs, suggesting that seven TFs (Ace2, Fkh1, Fkh2, Skn7, Stb1, Swi4 and Swi6) may have cooperativity with Mbp1 (Figure 3b). Among them, five PCTFPs (Mbp1-Fkh1, Mbp1-Skn7, Mbp1-Stb1, Mbp1-Swi4 and Mbp1-Swi6) are highly biologically plausible since they are supported by five types of validation information. The other two PCTFPs (Mbp1-Ace2 and Mbp1-Fkh2) are moderately biologically plausible since they are supported by four types of validation information.

**Figure 4.**
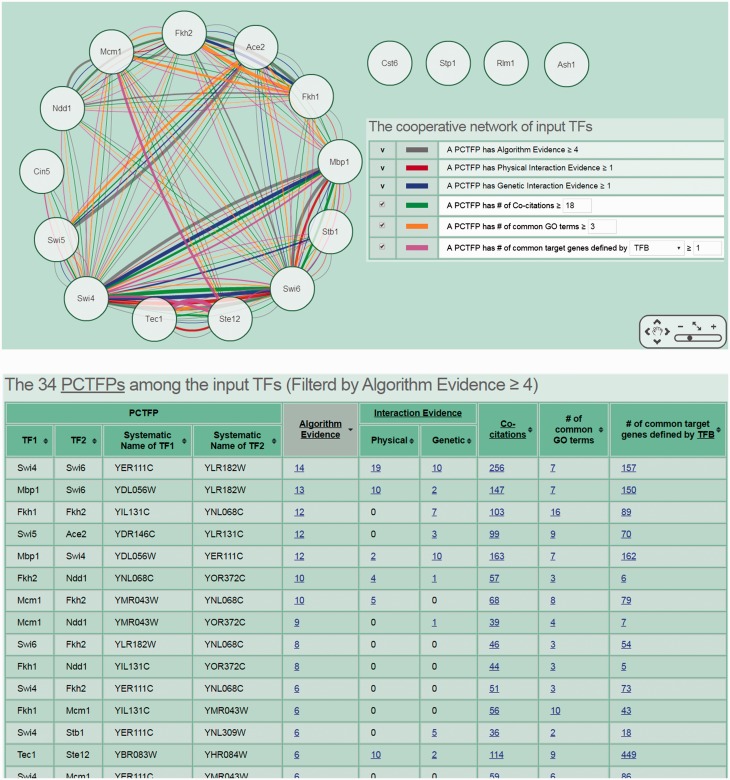
The second scenario of using CoopTFD. When users (i) select the first search function, (ii) input a list of 17 predicted cell cycle TFs and (iii) require that each PCTFP must be predicted by at least four algorithms, CoopTFD returns 34 PCTFPs. Among them, 18 PCTFPs are highly biologically plausible since they are supported by five types of validation information. The other 16 PCTFPs are moderately biologically plausible since they are supported by four types of validation information.

The second scenario is as follows. Researchers often have a set of genes of interest (e.g. differentially expressed genes under a specific biological condition) from microarrays. They then may use existing algorithms or tools to identify TFs that regulate this set of genes (32–34). If they also want to know possible PCTFPs among the identified TFs, they can use CoopTFD to do this task. For example, researchers can have a list of 17 predicted cell cycle TFs (Ace2, Ash1, Cin5, Cst6, Fkh1, Fkh2, Mbp1, Mcm1, Ndd1, Rlm1, Stb1, Ste12, Stp1, Swi4, Swi5, Swi6 and Tec1) from an existing algorithm (32). Now if they (i) select the first search function, (ii) input the list of 17 TFs and (iii) require that each PCTFP must be predicted by at least four algorithms, CoopTFD returns 34 PCTFPs (Figure 4). Among them, 18 PCTFPs are highly biologically plausible since they are supported by five types of validation information. The other 16 PCTFPs are moderately biologically plausible since they are supported by four types of validation information.

The third scenario is as follows. If researchers have already known key TFs of a specific biological process and would like to know the cooperative network of these TFs, they can use CoopTFD to do this task. Here, we use yeast oxidative stress response as an example. Yeast cells are constantly challenged by the oxidative stress which is induced by the reactive oxygen species (ROS). If not eliminated properly, ROS can damage all cellular components. Yeast cells respond to the oxidative stress by activating many genes involved in the oxidant defence mechanisms. Msn2, Msn4, Skn7 and Yap1 are key TFs which cooperatively regulate the expression the antioxidant genes (1). If users input these four TFs and require that each PCTFP must be predicted by at least two algorithms, then CoopTFD returns a densely connected cooperative TF network (consisting of six PCTFPs) among the four input TFs (Figure 2a and b). The provided cooperative TF network is likely to be biologically relevant since (i) all PCTFPs are predicted by at least two algorithms, (ii) 67% (4/6) PCTFPs have experimental evidence of having physical or genetic interactions, (iii) the two TFs of each of the six PCTFPs are studied in the same publications, (iv) all PCTFPs have common GO terms and (v) all PCTFPs have common target genes.

## Conclusion

In this article, we present CoopTFD which provides 2622 predicted cooperative TF pairs (PCTFPs) among 143 yeast TFs from 17 existing algorithms. By integrating multiple data sources, we also provide five types of validation information for each PCTFP to help users judge the biological plausibility of a PCTFP. The information includes the algorithms which predict a PCTFP, the publications which experimentally show that a PCTFP has physical or genetic interactions, the publications which experimentally study the biological roles of both TFs of a PCTFP, the common GO terms of a PCTFP, and the common target genes of a PCTFP. Using three scenarios, we show that CoopTFD can return biologically plausible PCTFPs of a TF or a biologically relevant cooperative network of a list of TFs. CoopTFD has an easy-to-use interface for biologists to search or browse for the PCTFPs of the TFs of interest. CoopTFD will be regularly updated based on the newly published literature and the latest releases of the BioGRID, SGD and YEASTRACT databases. We believe that the PCTFPs deposited in CoopTFD will be a very useful resource for yeast biologists to study the combinatorial regulation of gene expression by cooperative TFs.

## Supplementary Data

Supplementary data are available at *Database* Online.

Supplementary Data
