# Supplementary material for: CoopTFD: a repository for predicted yeast cooperative transcription factor pairs
Source: Database (Oxford). 2016 May 30;2016:baw092. doi: 10.1093/database/baw092 (PMC4885606; doi:10.1093/database/baw092)
Supplement: Supplementary Data [file supp_2016_baw092_index.html]

CoopTFD: a repository for predicted yeast cooperative transcription factor pairs — Supplementary Data 

# CoopTFD: a repository for predicted yeast cooperative transcription factor pairs

## Supplementary Data

files

- Supplementary Data - pdf file
