# Supplementary material for: CoopTFD: a repository for predicted yeast cooperative transcription factor pairs
Source: Database (Oxford). 2016 May 30;2016:baw092. doi: 10.1093/database/baw092 (PMC4885606; doi:10.1093/database/baw092)
Supplement: Supplementary Data [file supp_baw092_Supplementary_Figure_1.pdf]

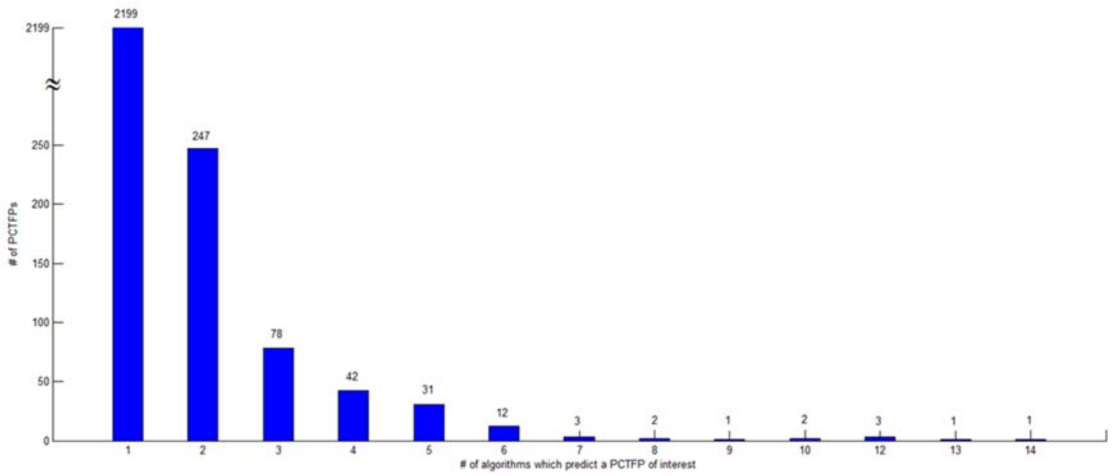

Supplementary Figure 1. This figure shows a distribution of numbers of PCTFPs against number of algorithms which predict a PCTFP of interest.
